# Manipulation of the Nasal Superficial Musculoaponeurotic System to Enhance Midvault and Supratip Contouring

**DOI:** 10.1093/asjof/ojae089

**Published:** 2024-10-24

**Authors:** Adam D Glener, Virginia E Bailey, Derek Sheen, Spencer Cochran

## Abstract

**Background:**

Management of dorsal dead space and the aesthetics of a supratip break are paramount to achieving reproducible and reliable results in rhinoplasty.

**Objectives:**

The authors present a modified technique of redraping the nasal soft tissue envelope in structural rhinoplasty by utilizing the nasal superficial musculoaponeurotic system (SMAS) to help obliterate dorsal dead space and restore normal anatomy, thereby enhancing midvault and supratip contouring.

**Methods:**

A standard open rhinoplasty approach is utilized. A planar transition from supraperichondrial to subperichondrial/subperiosteal is completed during the dorsal dissection. The open structural rhinoplasty then proceeds as previously published by the senior author. After any desired tip work is completed, the cephalically based SMAS layer is reconstituted with suture fixation laterally along the caudal border of the upper lateral cartilages. A more robust technical discussion is borne out in the manuscript.

**Results:**

At submission, the senior author has performed >100 rhinoplasties employing this technique over roughly 1 year. Subjectively, patients have had better immediate supratip contour with less residual dorsal soft tissue edema. A case example with photographs is included at a 1 year postoperative time point.

**Conclusions:**

Using this described technique, surgeons can employ an open, structural, approach to rhinoplasty while using restoration of natural anatomy to achieve a refined postoperative result, accentuating supratip break while minimizing dorsal soft tissue dead space.

**Level of Evidence: 5 (Therapeutic):**

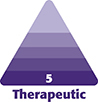

As the techniques for rhinoplasty have evolved over time, there has been a growing awareness of the significance and importance of preserving and restoring native nasal anatomy. To some degree, structural rhinoplasty has adapted to align with preservation rhinoplasty trends, emphasizing minimal soft tissue disruption and the preservation of desired anatomical features when feasible. Open structural techniques remain widely applicable and are particularly valuable in facilitating precise nasal tip contouring, enhancing visualization, and controlling graft placement.^1^ However, the advancement of preservation ideology has compelled even the most dogmatic structural rhinoplasty surgeons to consider the adoption of preservation techniques, even within a traditional structural setting. Although the majority of preservation techniques focus on the osseocartilaginous framework, the importance of preserving or reconstituting the soft tissue and ligamentous attachments cannot be overestimated. To this end, the authors present a modified technique of re-draping the nasal soft tissue envelope in structural rhinoplasty with specific attention to the obliteration of dead space and restoration of normal anatomy to enhance midvault and supratip contouring.

The soft tissue envelope of the nose is comprised of the skin, superficial fat, a fibromuscular layer (also known as the nasal superficial musculoaponeurotic system or SMAS), a deeper fat/muscle layer, and nasal skeletal structures.^2^ The nasal SMAS, identified as early as the 1980s, and reaffirmed in subsequent anatomical studies, plays a crucial role in separating the superficial nasal tissues from the underlying osseocartilaginous framework.^2-4^ The significance of this layer becomes evident when disrupted, as the dermis has a higher propensity to adhere to the underlying nasal skeleton, ultimately causing contour deformity and fibrotic thickening.^3^ Furthermore, failure to reconstitute the SMAS layer can lead to remote distortion of the cartilaginous framework because of the contractile forces of the healing dermis.^3^ Lastly, maintaining an intact SMAS layer has implications on the postoperative course; a sub-SMAS dissection decreases postoperative edema, limiting supratip fullness and loss of definition.

## METHODS

The study was performed according to the guiding principles of the Declaration of Helsinki. The study was IRB exempt under 45 CFR § 46.104(d)(4) (WCG IRB, Puyallup, WA).

**Figure 1.**
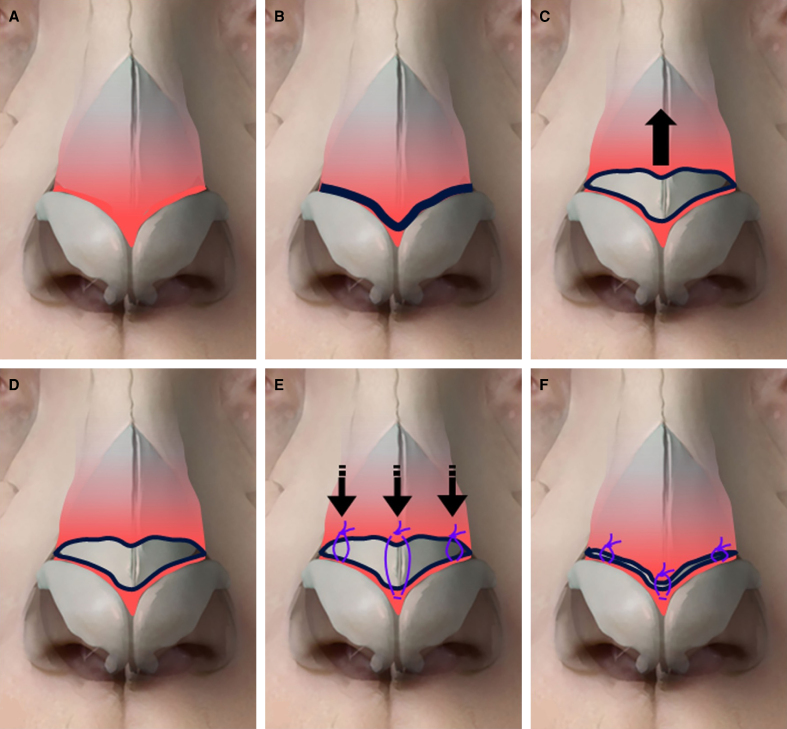
Illustration of manipulation of the nasal superficial musculoaponeurotic system (SMAS). (A) The SMAS layer (depicted in red) in situ. (B) The level (marked in black) that the SMAS is incised, just caudal to the lower lateral cartilages. (C) The cephalic retraction that occurs once the SMAS is incised, also demonstrated in Video 1. (D) The SMAS in its cephalically retracted position. (E) The pattern and position of fixation sutures. (F) The SMAS in its reconstituted, anatomic position.

**Figure 2.**
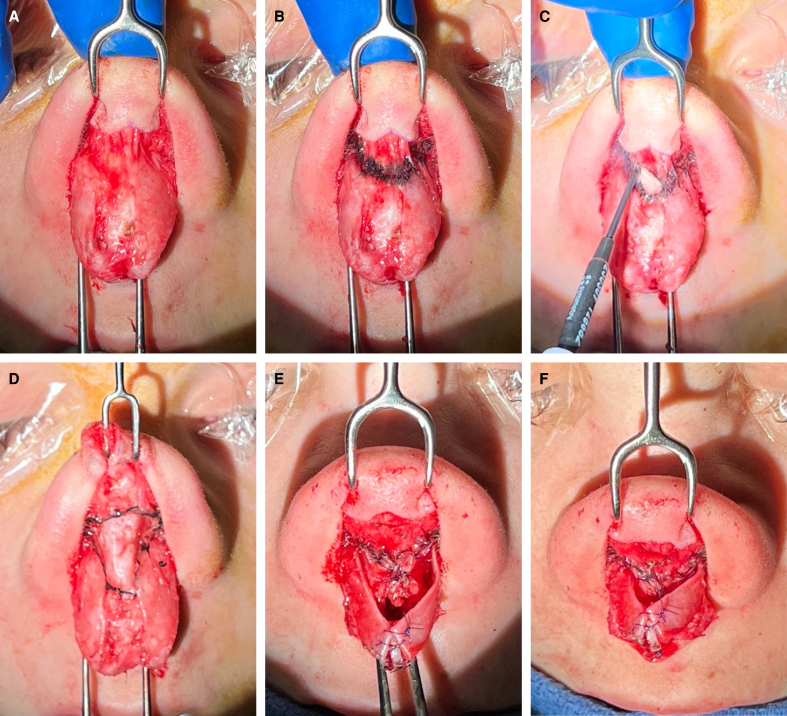
Surgical demonstration of nasal superficial musculoaponeurotic system (SMAS) manipulation, coinciding the depiction presented in Figure 1. (A) The SMAS layer shown in situ. (B) The level (marked in black) that the SMAS is incised, just caudal to the lower lateral cartilages. (C) The cephalic retraction that occurs once the SMAS is incised, also demonstrated in Video 1. (D) The SMAS in its cephalically retracted position. (E) The pattern and position of fixation sutures with the lower lateral cartilages caudally retracted for visualization. (F) The SMAS in its reconstituted, anatomic position.

**Figure 3.**
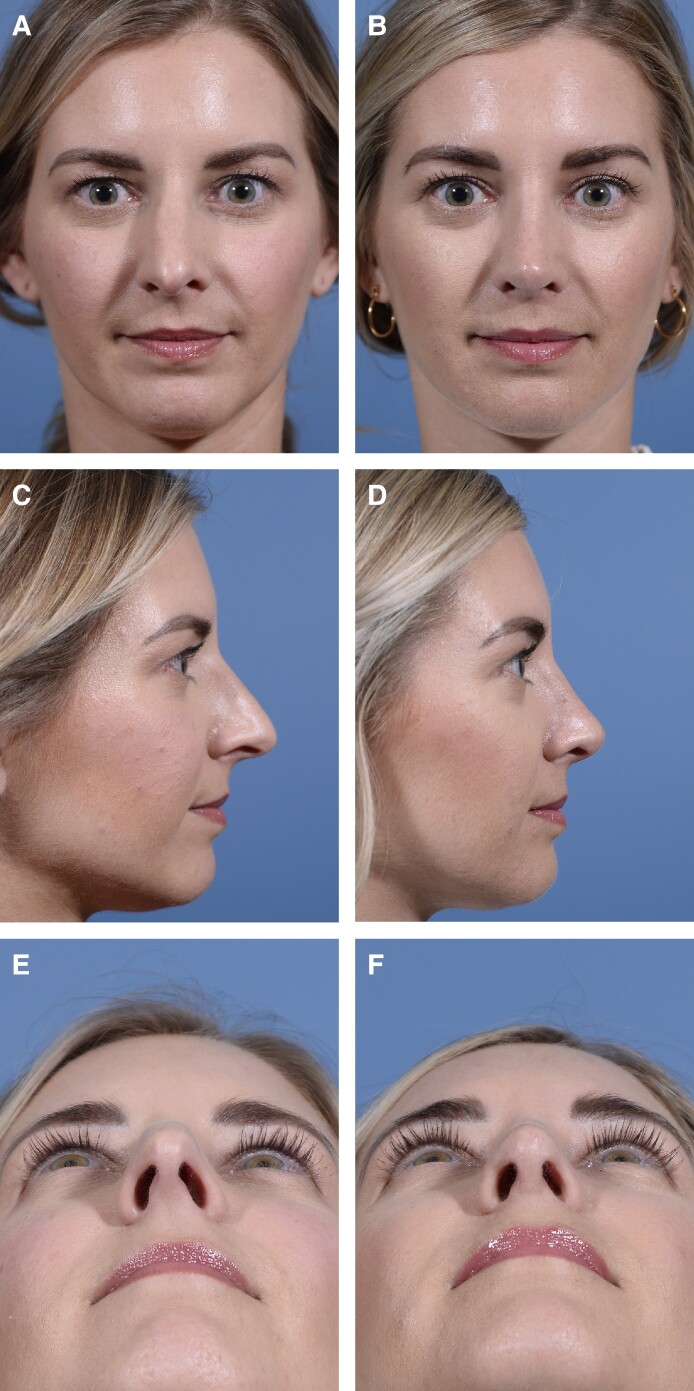
Preoperative and 1 year postoperative images of a 32-year-old female are presented in the anteroposterior (A, B), lateral (C, D), and basal (E, F) views.

The surgical technique is described below and illustrated in Figures 1 and 2. An open rhinoplasty approach is performed through a transcolumellar incision, and the lower cartilages are exposed as the soft tissue envelope is elevated from the lower lateral cartilages in the sub-SMAS supraperichondrial plane. Along the caudal edge of the upper lateral cartilage, and cephalic to the intercrural ligament, monopolar cautery is then used to transition from the supraperichondrial to the subperichondrial plane overlying the cartilaginous midvault; this transition point allows for the preservation of the scroll ligaments. Although cephalic to their insertion, this transition does manipulate the vertical components of the scroll ligament; understanding of the ligamentous anatomy in this area is important.^5,6^ During this planar transition, an investing portion of the nasal SMAS is divided and inherent tension is released, as evidenced by the immediate cephalic retraction of the SMAS layer (Video 1). The caudal edge of the SMAS layer is delineated with a marking pen for later reconstitution at the caudal edge of the midvault. The open structural rhinoplasty then proceeds in the previously described sequence of events.^7-9^ After any desired tip work is completed, the previously marked and cephalically based SMAS layer is reconstituted with suture (6-0 Polydioxanone) fixation laterally along the caudal border of the upper lateral cartilages and medially to the septal angle (Video 2). This pulls the retracted SMAS layer inferiorly and progressively tensions the overlying soft tissues, enhances midvault definition, and reduces supratip and midvault soft tissue bulk. Figure 3 illustrates a patient 1 year after undergoing open structural rhinoplasty using the above technique.

## RESULTS

As is common with many technical advances in plastic surgery, this technique was devised to address long-term defects. The senior author anecdotally noticed a subset of postoperative patients presenting with persistent midvault fullness that was refractory to steroid injections. These refractory patients all had a palpable soft tissue band just cephalic to the supratip break, a phenomenon similarly described by Daniel and Kosins.^10^ Therefore, moving forward, the senior author has reconstituted this SMAS layer as described above, having completed >100 open rhinoplasties using this technique. Thus far, reconstituting the nasal SMAS has resulted both in a more refined supratip break immediately postoperatively and, to this date, avoidance of the midvault fullness resulting from the retracted SMAS layer. Anecdotally, the patient has had less dorsal soft tissue edema with a faster recovery. A patient is depicted in Figure 3 preoperatively and then at 1 year after surgery with the described technique.

## DISCUSSION

Manipulation of the nasal SMAS was recently described by Cobo in 2018 with similar noted refinement of the supratip break in the immediate postoperative period.^11^ Cobo describes developing the dermis and underlying SMAS in 2 distinct soft tissue flaps and insetting the SMAS flap between the caudal medial crura.^11^ Our technique described here has been adapted to elevate the SMAS and overlying dermis as a composite flap, thereby preserving the subdermal plexus and ensuring vascular integrity while minimizing dorsal dead space and decreasing edema from excessive soft tissue dissection and disruption of subdermal lymphatics. Additionally, the authors feel that reconstitution of the SMAS flap at its original point of division at the scroll ligament (and not transposing it to re-drape the tip as described by Cobo) allows for a refined reconstruction of the underlying anatomy with the potential benefit of tensioning the internal nasal valve laterally. As discussed in the preservation literature, reconstitution of the supratip anatomy creates a seamless transition of soft tissue from the midvault to the nasal tip, stabilizes the internal nasal valve, and accentuates the supratip break.^10^

As with any surgical technique, there are some limitations and technical nuances when employing this maneuver. If the suture captures any dermis, instead of just SMAS, the result can be soft tissue dimpling or even button-holing. It is imperative that when resecuring the SMAS, only the SMAS layer from the dorsal flap is included in the fixation. Additionally, the sutures should not be overtensioned—this will result in an overly accentuated supratip break, especially in thinner skinned patients (Video 2 demonstrates tensioning of the SMAS re-inset). This technique reconstructs the intrinsic anatomy and thereby accentuates supratip break; if overdone, it would certainly affect dorsal aesthetics. As this is a novel technique for the senior surgeon, there are limited numbers of patients with long-term follow-up available; although the senior author has utilized this technique in over 100 rhinoplasties over the past year of adoption. Additional data are certainly warranted, including, possibly, another study where the supratip aesthetics are compared in patients who underwent this technique and those who predated the adaptation.

## CONCLUSIONS

The authors believe that structural and preservation rhinoplasty should not be mutually exclusive; instead, elements from both techniques can be combined to enhance the strength of each other. Using this described technique, surgeons can employ an open, structural approach to rhinoplasty while using restoration of natural anatomy to achieve a refined postoperative result.
